# Fighting Drug Resistance through the Targeting of Drug-Tolerant Persister Cells

**DOI:** 10.3390/cancers13051118

**Published:** 2021-03-05

**Authors:** Giulia De Conti, Matheus Henrique Dias, René Bernards

**Affiliations:** Division of Molecular Carcinogenesis, Oncode Institute, The Netherlands Cancer Institute, Plesmanlaan 121, 1066 CX Amsterdam, The Netherlands; g.d.conti@nki.nl (G.D.C.); m.dos.santos.dias@nki.nl (M.H.D.)

**Keywords:** drug tolerance, cellular plasticity, adaptive resistance, collateral vulnerability, stress response

## Abstract

**Simple Summary:**

A major obstacle in the fight against cancer is the development of drug resistance in response to therapy. Development of resistance can be facilitated by a small population of drug-tolerant cancer cells characterized by an increasead capacity of adaptation to various stresses. Here, we describe features and vulnerabilities of these drug-tolerant persister cells that can be exploited to prevent the development of drug resistance.

**Abstract:**

Designing specific therapies for drug-resistant cancers is arguably the ultimate challenge in cancer therapy. While much emphasis has been put on the study of genetic alterations that give rise to drug resistance, much less is known about the non-genetic adaptation mechanisms that operate during the early stages of drug resistance development. Drug-tolerant persister cells have been suggested to be key players in this process. These cells are thought to have undergone non-genetic adaptations that enable survival in the presence of a drug, from which full-blown resistant cells may emerge. Such initial adaptations often involve engagement of stress response programs to maintain cancer cell viability. In this review, we discuss the nature of drug-tolerant cancer phenotypes, as well as the non-genetic adaptations involved. We also discuss how malignant cells employ homeostatic stress response pathways to mitigate the intrinsic costs of such adaptations. Lastly, we discuss which vulnerabilities are introduced by these adaptations and how these might be exploited therapeutically.

## 1. Introduction

In the last decades, targeted therapies, as well as immunotherapeutic approaches, have been developed with the promise of revolutionizing cancer care. However, despite the undeniable improvement in patient prognosis delivered by these new therapies, the development of drug resistance remains a major obstacle in cancer treatment. Drug resistance can be categorized as either primary resistance, when the treatment has no objective effect on cancer cells from the start, or as secondary resistance, in which unresponsiveness follows an initial response. The development of acquired drug resistance is intimately linked to intratumor heterogeneity in terms of genetic, epigenetic, transcriptional and metabolic state. Intratumor genetic heterogeneity has been demonstrated extensively by genome sequencing efforts in solid and hematological tumors, shedding light on the complex clonal composition and dynamics of the tumor [1]. However, most of the available information regarding this evolution has been acquired through bulk tumor DNA sequencing or sequencing of multiple biopsies from the same patient. The development of single-cell sequencing (sc-seq) has enabled a more detailed characterization of intratumor heterogeneity both at a genetic and a non-genetic level. Moreover, scRNAseq approaches allow an investigation of the dynamic changes in cellular signaling pathways in response to therapy. 

**Figure 1:**
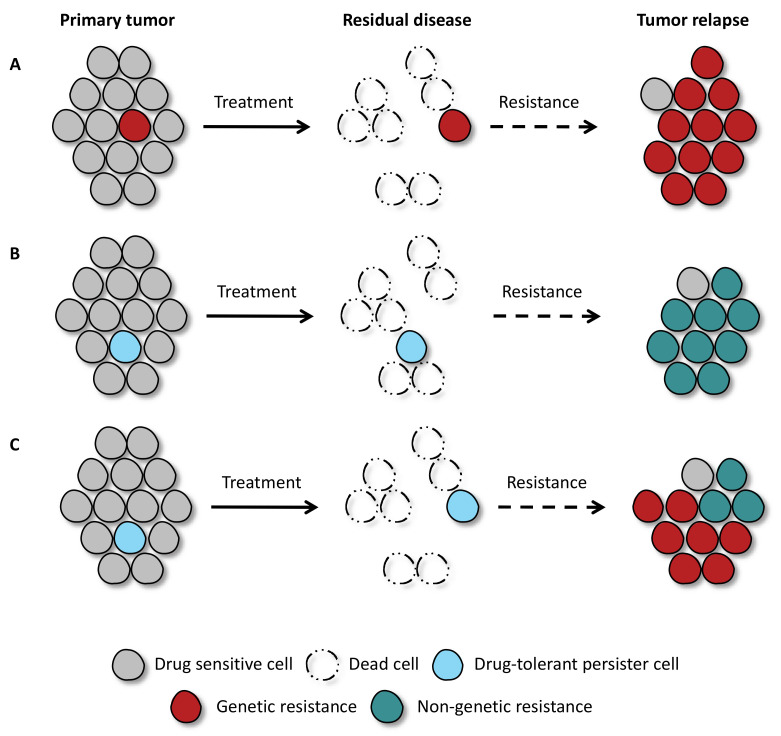
Cancer cells can acquire drug resistance via genetic and non-genetic adaptation mechanisms. Cancer cells initially respond to drug exposure. However, few malignant cells can survive the treatment. (**A**) Pre-existing clones, with specific genetic mutations, are intrinsically resistant to targeted therapy and allow for the tumor to regrow. (**B**) In the absence of pre-existing genetic mutations, few drug-tolerant persister cells are spared during drug treatment, and over time, these cells can give rise to tumor relapse. (**C**) From the pool of drug-tolerant persister cells, different genetic and non-genetic resistance mechanisms can emerge and contribute to tumor regrowth.

Acquired drug resistance has been attributed largely to the selection or de novo acquisition of specific gene mutations that confer proliferative and/or survival advantage to escape the therapeutic pressure (Figure 1A). However, it has become evident that drug resistance can also be achieved without a specific genetic event (Figure 1B). 

Genetic and non-genetic adaptations during the acquisition of drug resistance are not mutually exclusive (Figure 1C). It has been suggested that non-genetic adaptations provide initial survival under therapy, allowing a small population of tumor cells to acquire secondary resistance mutations, resulting in disease progression [2]. Such a population of residual malignant cells that are spared by the treatment is defined clinically as “minimal residual disease” (MRD). These residual cells form a reservoir from which fully resistant tumor cells can emerge. Using barcoding technology, it has been shown that genetic resistance to therapy is often pre-existing in a tumor population [3]. This has also been demonstrated in a longitudinal study of B-cell Acute Lymphoblastic Leukemia (B-ALL) patient-derived samples, in which relapse initiating clones were present as minor latent subclones at diagnosis and subsequently selected by chemotherapy [4]. If the above-mentioned model of sequential non-genetic adaptation followed by genetic mutation is correct, then the barcoding study also implies that the persistent population pre-exists in the tumor prior to therapy. 

Operationally, these cells are defined as drug-tolerant persister (DTP) cells. They represent a rare and noncycling or slowly cycling population of cells in the presence of the drug, characterized by altered metabolism, distinct epigenetic features and transcriptional program. In vitro, they can be generated by exposing tumor cells to cytotoxic concentrations of therapeutically relevant drugs [5]. The vast majority of cancer cells are killed, but this small fraction of drug-tolerant cells appears with different kinetics and frequencies, depending on the cancer type and the drug. 

In vitro, after drug withdrawal, DTPs can resume proliferation, but their progeny remains equally sensitive to the initial therapy, indicative of a transient and not an inheritable resistance mechanism [5,6]. The first report demonstrating the transient acquisition of a drug-tolerant persister phenotype at a low frequency used *EGFR*-mutant non-small cell lung cancer (NSCLC) cells treated with gefitinib. DTPs generated by gefitinib treatment appeared at a very low frequency (about 0.3%) and were characterized by extensive epigenetic alterations rather than any genetic alteration [5]. Similarly, in a *BRAF^V600E^* melanoma model, treatment with vemurafenib was shown to upregulate *EGFR* expression to overcome the mitogen-activated protein kinase (MAPK) pathway inhibition in response to drug treatment. However, when the drug is removed, the supraphysiological level of MAPK activation induces a state of oncogene-induced senescence, and cells expressing high levels of EGFR are counter selected. This, in turn, leads to the restoration of vemurafenib sensitivity [7]. In line with this observation, the combination of BRAF and EGFR inhibitors has been proved to be effective in *BRAF^V600E^* colon cancer patients [8,9]. 

Clinical evidence also indicates that, after initial progression under treatment, patients can respond to the rechallenge with the same therapy following a “drug holiday” [10]. Such disease remission suggests a reversible and adaptive response to the drug. Nonetheless, rechallenging relapsed patients with the same targeted therapy is rare in clinical practice due to the observation that most tumors are no longer responsive to the initial therapy upon rechallenge, indicative of a stable form of resistance in most cases [11,12]. However, for many cases, there is a lack of a clear genetic explanation for this stable, inherited drug resistance in both hematological (low mutation burden) and solid cancers (high mutation burden). Thus, in contrast to the reversible non-genetic resistance seen in DTPs, it appears that some non-genetic resistance mechanisms can also be stably inherited. 

**Figure 2:**
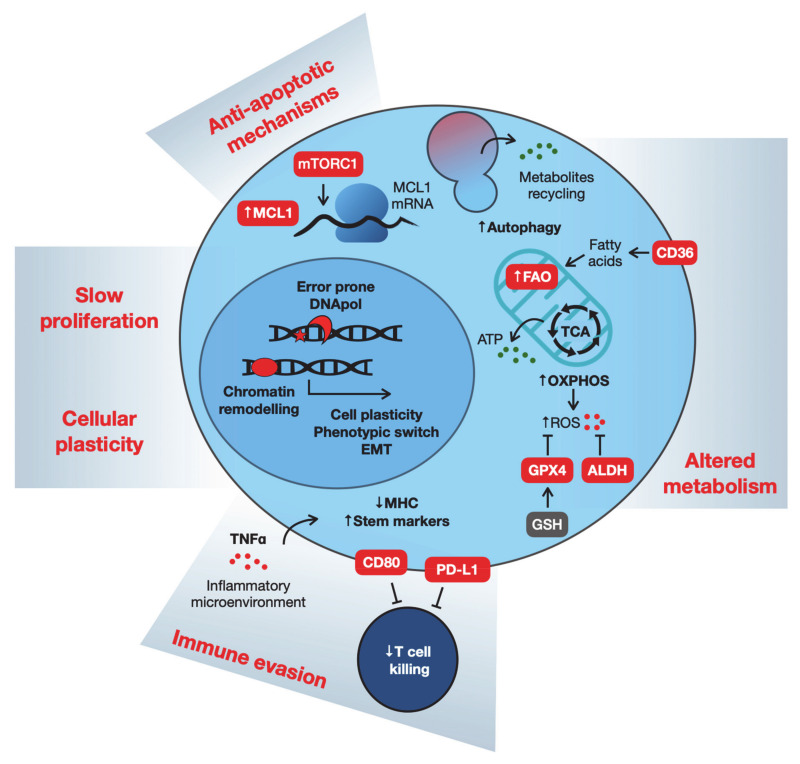
Features and vulnerabilities of drug-tolerant persister cells. Specific characteristics of drug-tolerant persister (DTP) cells are linked to the development of vulnerabilities that, in turn, can be exploited to eliminate them. Persister cells are characterized by deep transcriptional reprogramming, allowing survival in the presence of cytotoxic drugs. Transcriptional changes enable the slowdown of DTP proliferation and favor the upregulation of error-prone DNA polymerase. DTPs show altered metabolism, becoming more dependent on oxidative phosphorylation and antioxidant mechanisms. Alternative sources of nutrients can be obtained by upregulating autophagy and fatty acid oxidation. Lastly, cellular plasticity allows DTPs to evade immune-mediated clearance, and the upregulation of anti-apoptotic mechanisms protects persister cells from cytotoxic drugs.

There is still no clear consensus about characterization, markers or even the origin of the different slowly proliferating drug-tolerant phenotypes. Nonetheless, they share common features such as a slow cell cycle, altered metabolism, altered epigenetic features, resistance to apoptosis and immune evasion. Underlying all these features is the increased cellular plasticity and heterogeneity that provides cells with multiple possibilities to rewiring cellular signaling to evade cytotoxic therapies (Figure 2). Below, we first review some relevant data on these features common to drug-tolerant phenotypes. Subsequently, we discuss how homeostatic stress response pathways are mobilized to support the drug-tolerant state. We conclude by discussing different strategies to target drug-tolerant cancer cells to suppress the emergence of acquired drug resistance. 

## 2. What Are the Characteristics of Drug-Tolerant Cells?

### 2.1. Cell Cycle Restriction

Multiple definitions of cell cycle-restricted drug-tolerant cancer cells are used, associated with different contexts. For instance, dormant cancer cells are defined as rare cells that can disseminate early and stay latent in distant niches before being reactivated and cause disease relapse after successful treatment of the primary tumor [13]. Therefore, in the clinical setting, the term dormancy is used to refer to the time between treatment of the primary tumor and metastatic relapse at secondary sites. An apparent difference between DTPs and disseminated dormant cancer cells is that the latter can originate from the primary tumor prior to any therapeutic intervention so that their phenotype is not necessarily linked to drug tolerance [14]. Despite these differences in origin, it remains to be addressed which features are shared by DTPs and disseminated dormant cells. 

Since DTPs are characterized by a reversible restriction of proliferation, they resemble quiescent cells. Quiescence is a physiological cell cycle state (G0) prevalent in adult stem cells from tissues with low turnover but also a characteristic of cancer stem cells (CSCs). Even though the relationship between CSCs and DTPs is still not completely clarified, some similarities become evident. For instance, increased tolerance to therapies is also a common characteristic of CSCs (reviewed in [15]), while stem-like features have been described in DTPs from several cancer types [5]. In hematological malignancies, it has been extensively demonstrated that, interfering with CSC biology, sensitizes tumors to chemotherapy. A recent paper identified calcitonin receptor-like receptor (CALCRL) is highly expressed in leukemia DTPs and its depletion reduces leukemia stem cell frequency after chemotherapy [16]. Consistent with this notion, cancers with high expression of stemness gene signatures are associated with poor prognosis [17,18]

Perhaps at the end of the “spectrum” of cell cycle-restricted phenotypes, senescence is defined as a stable cell cycle arrest. Senescence induction is the physiological cellular response to telomeres shortening, DNA damage, excessive oncogenic signaling or a variety of other stress signals [19]. Interestingly, radio- and chemotherapies are able to induce cancer senescence, the so-called therapy-induced senescence (TIS) [20]. Senescent cancer cells share common features with normal senescent cells, such as the formation of heterochromatic foci (SAHF), the production of a plethora of secreted factors (SASP) and the resistance to apoptotic cell death. Importantly, it has been shown recently that senescent cancer cells maintain the ability to re-enter the cell cycle in response to changes in the tumor microenvironment or epigenetic alterations. In particular, Schmitt and colleagues described the enhanced tumor-initiating potential of lymphoma cells that escaped senescence [21]. These observations identify senescence as a potential drug-tolerance mechanism to support acquired resistance. 

Very recently, two groups identified a state of drug adaptation that resembles “diapause”, a delay in embryonic development that can occur under stress conditions [22,23]. One remarkable feature of these diapause-like cells was that there did not appear to be a loss of clonal complexity of tumors that went through this DTP-like state. This is inconsistent with the finding of others that DTPs are typically a very small subset (0.3–5% of cells) of the entire tumor population [5,24]. It is therefore not clear at present whether this diapause-like state is fundamentally different from the less frequent DTPs. Single-cell RNAseq experiments may shed light on the differences and similarities of the different DTP populations. 

In these diapause-like DTP models, the suppression of MYC activity was sufficient to induce the state of drug tolerance, pointing toward a close link between proliferation state and drug tolerance. Consistent with this, it has been proposed previously that the slow proliferation rates common to the phenotypes described above provide a survival advantage in the presence of cytotoxic drugs [25,26]. More recently, this cell cycle restriction was also associated with the upregulation of error-prone DNA repair mechanisms that can favor the development of full resistance [2]. This scenario suggests that slow-proliferating drug tolerance may be a key intermediate step toward acquired genetic resistance. 

### 2.2. Metabolism

Cellular plasticity allowing changes in chromatin state and transcription modulation also impact the overall metabolism of slow-proliferating drug-tolerant phenotypes. DTP cells have been described to slow down their metabolism, becoming less dependent on glycolysis and increasingly dependent on mitochondrial oxidative phosphorylation (OXPHOS). The upregulation of this pathway has been shown in models of *KRAS^G12D^* pancreatic ductal adenocarcinoma (PDAC) [27], *BRAF^V600E^* melanoma [25], as well as Acute Myeloid Leukemia (AML) [28] and Chronic Myeloid Leukemia (CML) [29], upon treatment with targeted- or chemotherapies. As a result of the increased OXPHOS activity, persister cells become more oxidatively stressed. A commonly upregulated pathway activated as an antioxidant response is the glutathione-dependent reduction of lipid peroxides, and recent studies identified phospholipid glutathione peroxidase 4 (GPX4) inhibition as a potent strategy to eradicate DTP cells via the induction of ferroptosis [30,31]. Interestingly, aldehyde dehydrogenase (ALDH), a known stem cell marker, has also been described to protect persister cells from reactive oxygen species (ROS)-mediated toxicity [32]. Moreover, NRF2, a master regulator of redox homeostasis, is tightly regulated in DTP cells to promote oxidative stress tolerance [30,33]. It is noteworthy, however, that increased OXPHOS activity is not a universal feature of DTPs. In a Eµ-Myc-driven mouse lymphoma model, persister cells tolerant to chemotherapy displayed a very active metabolism, with increased glucose consumption despite their senescent phenotype [34].

Another common feature of DTP cells is the ability to sustain their metabolism with alternative sources of nutrients such as autophagy or fatty acid oxidation (FAO) pathways [27,35,36]. Autophagy allows metabolites recycling derived from the degradation of macromolecules and, together with the production of acetyl-CoA in the FAO process, sustain ATP production in the mitochondria. Increased FAO is required for *BRAF^V600E^* melanoma cells to survive MAPK inhibition before the development of drug resistance [37,38]. In the same model, CD36, a fatty acid transporter is upregulated in response to MAPK inhibition, and it is a useful marker of melanoma cells during adaptation and drug-tolerant phases. Interestingly, CD36 is also essential for the survival of HER2-positive breast cancers during targeted HER2/EGFR inhibition, therefore being associated with non-genetic resistance to therapy [39]. 

### 2.3. Anti-apoptotic Mechanisms

Overall metabolism and protein production in human cells are mostly regulated according to proliferation states [40]. Consistently, slow-proliferating drug-tolerant cells are characterized by a low level of mRNA translation. Recent studies also describe a role for post-transcriptional modification to enhance the stability of specific mRNAs involved in prosurvival signals [6,41]. Resistance to apoptosis is a prerequisite for drug resistance, but it is not clear whether it is primarily a defective apoptotic process that allows the population of persister cells to survive drug treatment. The link between DTPs and apoptosis resistance was underscored by the finding that DTPs from *EGFR*-mutated NSCLC can survive gefitinib and osimertinib treatments by upregulating the anti-apoptotic protein MCL1 via the mTORC1-mediated post-transcriptional regulation of mRNA translation [42]. Understanding of the dynamics of apoptotic pathways in DTPs is particularly interesting because a number of drugs have been developed to target anti-apoptotic proteins, and several clinical trials are ongoing in hematological and solid cancers. Moreover, drug-induced senescent cancer cells have an increased sensitivity to one of these drugs, ABT263 (navitoclax), a specific inhibitor of the anti-apoptotic proteins BCL-2, BCL-W and BCL-XL [43].

### 2.4. Immune Evasion

It is still understood poorly whether persister cells are also at the basis of adaptive resistance to immunotherapies. However, a recent study described the emergence of a discrete subpopulation of cells resistant to an immune-checkpoint inhibitor (anti-PD1 antibody) in a murine organotypic spheroid ex vivo model [44]. These cells were resistant to CD8+ T cell-mediated killing and expressed Snai1 and the stem cell antigen Sca1. Interestingly, these persister cells rely on Birc2/3 anti-apoptotic factors, and their inhibition, combined with anti-PD1 therapy, enhanced tumor cell killing in vivo [44]. In response to anti-PD1 treatment, melanoma patients acquired resistance via the downregulation of major histocompatibility complex class 1 (MHC-I), associated with TGFß activity, SNAI1 upregulation and a dedifferentiated phenotype [45]. Adaptive resistance to immunotherapy is associated with the upregulation of alternative immune-checkpoint molecules [46]. Similarly, the interaction between the immune system and dormant cancer cells has been studied extensively and mechanisms of immune evasion include the downregulation of MHC-I molecules [47] and the upregulation of PD-L1 [48]. 

In response to adoptive T cell transfer (ACT), the tumor microenvironment becomes rich in inflammatory mediators. In particular, TNFɑ has been identified to induce the reversible loss of antigens in melanoma cells, preventing their killing [49,50]. Similarly, in a model of squamous cell carcinoma, ACT was able to clear the bulk of the tumor, but few slow-cycling cells were refractory. These cells acquired the expression of CD80, a ligand of the cytotoxic T lymphocyte antigen-4 (CTLA4), dampening the cytotoxic T cell activity [51]. 

Therapeutic interventions can also indirectly favor DTPs immune evasion. In response to T cell therapy, the inflamed stromal cells secrete hepatocyte growth factor (HGF) and favor the recruitment of immune-suppressive neutrophils that restrain T cell expansion and function [52]. The recruitment of immune-suppressive cells was also described in models of breast cancer: after targeted inhibition, residual tumor cells expressed an inflammatory program driven by TNF⍺/NFkB signaling. In particular, high levels of CCL5 recruited immune-suppressive macrophages, which, in turn, favored tumor recurrence [53].

Moreover, multiple reports, both in solid and hematological cancers, highlighted the fundamental role of DTP cell plasticity in favoring immune evasion. 

### 2.5. Cellular Plasticity

The enhanced cellular plasticity of cancer cells facilitates the non-genetic adaptations underlying drug tolerance reviewed above. Such plasticity involves changes in the transcriptional program of the cells, with various intracellular pathways potentially merging into epigenetic changes and modulation of transcriptional factors to dictate cell behavior [5,25]. For instance, in response to targeted therapy, DTP melanoma cells exhibited a neural crest stem cell transcriptional state that favors the development of fully resistant clones [54]. Changes in the chromatin organization also modulate transcriptional programs to adapt to drug pressure. This process can result in phenotype switch such as the epithelial-to-mesenchymal transition (EMT) or in the acquisition of stem/progenitor-like phenotypes. EMT has been associated with the early survival of DTP cells in *EGFR*-mutated NSCLC upon tyrosine-kinase inhibition (TKI). Mesenchymal cells from NSCLC biopsies have been used as surrogate of persister cells to identify FGFR inhibition as a promising combination therapy to prevent DTP survival and expansion [55]. In triple-negative and basal-like breast cancers, DTP cells with altered expression of differentiation-state markers emerge during treatment with MEK and PI3K/mTOR inhibitors via a cell state transition involving chromatin remodeling [56]. Changes in chromatin organization have also been reported in basal cell carcinoma. In response to the Hedgehog pathway inhibitor vismodegib, a quiescent residual tumor population is selected. These cells express a transcriptional program similar to skin stem cells, enabled by a more permissive chromatin state and WNT pathway activation [57,58]. Cellular plasticity can also facilitate immune evasion. Neuroendocrine transformation of *EGFR*-mutant lung adenocarcinoma in response to erlotinib results in reduced expression of MHC-I complex and loss of tumor antigen presentation [59]. 

Longitudinal scRNAseq analysis of lung cancer patient biopsies, before and during target therapy, identified, at MRD, cells expressing an alveolar-regenerative signature, suggesting a therapy-induced cell plasticity [60]. Remarkably, this study described how not only cancer cells but also tumor-microenvironment adaptation to drug treatment influences clinical outcomes. 

## 3. Homeostatic Stress Response Pathways Support Slow-Proliferating Drug Tolerance

Genetic and epigenetic alterations leading to oncogenic signaling confer onto cancer cells the capabilities summarized as the hallmarks of cancer [61], as well as the increased cellular plasticity supporting drug tolerance reviewed above. However, cancer cells often activate stress response pathways to counterbalance the non-physiological oncogenic signaling to maintain cellular homeostasis [62]. This feature has been described in the context of in vitro malignant transformation [63], experimental in vivo tumor models [64], patient tumor samples [65] and metastasis [66]. A rearrangement of the balance between oncogenic signaling and stress response pathways may explain why drug-resistant cells survive under the stress induced by the drug. This implies that interfering with the stress response may be an Achilles heel of stressed cancer cells. For instance, inhibition of the DNA damage response (DDR) has been shown to sensitize cancer cells to chemotherapy and radiotherapy [67,68,69]. Likewise, in the context of targeted therapies, increased oxidative stress is observed in melanoma cells resistant to BRAF inhibition [70], while autophagy supports drug resistance against EGFR-targeted therapies in EGFR-driven cancer cells [71]. If the capability of cancer cells to deal with the oncogenic state and additional perturbations is critically dependent on stress response signaling, it may also be the case in the transition from drug tolerance to full-blown resistance. A rewiring of oncogenic signaling and stress pathways may be a key step to induce and sustain drug-tolerant states that allow bona fide resistance mechanisms to emerge. Indeed, autophagy [72], altered redox metabolism [73], replication stress response [74] and unfolded protein response (UPR) activation [75] have been individually shown to support the viability of cells with slow-proliferating drug-tolerant phenotypes in different models. Not surprisingly, this balance has been recognized increasingly as a source of potential therapeutic targets to forestall drug resistance.

The specific triggers and molecular mechanisms by which slow-proliferating drug-tolerant cancer cells engage these stress pathways are variable and so are the vulnerabilities associated with the different DTPs. For instance, autophagy has been shown to tame excessive oxidative stress, supporting the survival of dormant metastatic breast cancer cells by “eating” damaged mitochondria, with little effect in proliferating metastatic cells. Autophagy inhibition prevents the dormancy-to-growth switch and induces apoptosis in this model [72]. A stem cell-like population of nasopharyngeal carcinoma cells was shown to become radioresistant by the MYC-driven upregulation of CHK1 and CHK2 and consequent increased activation of DNA-damage-checkpoint responses. The knockdown of MYC or CHK1/2 resensitizes these cells to ionizing radiation [76]. Furthermore, p38 activation in dormant human carcinoma cells upregulates BiP and activates PERK, a component of the UPR. In these cells, the mobilization of those endoplasmic reticulum (ER) stress pathway components promotes resistance to etoposide, doxorubicin and ER stressors by inhibiting BAX-induced apoptosis. After the inhibition of PERK or knockdown of BiP, doxorubicin effectively kills these cells [75]. Protective UPR was also shown to facilitate tolerance to EGFR inhibitors in NSCLC cells. In this case, the disruption of ufmylation activates the IRE1α arm of the UPR, promoting BCL-XL-mediated cell survival. BCL-XL inhibition or further induction of ER stress with tunicamycin effectively targets these persister cells [77]. This set of data highlights how the phenotypic plasticity characteristic of cancer cells allows a variety of possibilities to rewire stress signaling pathways in order to support slow-proliferating drug-tolerant cells. 

The rewiring of mitogenic and survival pathways has also been proposed to support the viability of cells having slow-proliferating drug-tolerant phenotypes, creating additional targetable vulnerabilities. Using EGFR-driven NSCLC models, Sharma and colleagues showed that treatment with gefitinib or cisplatin results in a population of DTPs that have a repressed chromatin state induced by an upregulated IGF-1R signaling, whose inhibition is lethal to these cells [5]. The IGF1/IGF-1R signaling axis was also shown to promote the survival of dormant pancreatic cancer cells after oncogenic KRAS or MYC ablation in pancreatic cancer cells [78]. In a breast cancer model, the inhibition of SRC kinase is able to prevent the outgrowth of dormant cells in vitro and in vivo but does not induce cell death. The concomitant inhibition of MEK1/2 induces apoptosis and reduces the metastatic burden in the lungs [79]. It is also becoming increasingly evident that there is a role for the tumor microenvironment in regulating the survival of DTPs. In a model of metastatic prostate cancer, the contact with osteoblasts present in the bone marrow niche induces the expression of TBK1 leading to mTOR inhibition, dormancy, and drug resistance in the tumor cells. TBK1 knockdown reverts this stem-like phenotype and sensitizes prostate cancer cells to taxane treatment [80]. The data above emphasize that multiple prosurvival pathways can also be overridden to maintain the homeostasis of slow-proliferating drug-tolerant cells and promote a later dormancy-to-proliferation switch.

## 4. Targeting Slow-Proliferating Drug-Tolerant Cells

Given the dynamic nature of the slow-proliferating drug-tolerant phenotypes, the timing of targeting may be critical. This adds one more layer to the already complex nature of the interplay between oncogenic prosurvival signaling and stress response pathways. While some alterations in prosurvival signaling or stress response pathways may be transient and related to the slow-proliferating drug-tolerant phenotype, others can be maintained after full-blown resistance is established. Ideally, vulnerabilities of DTPs should persist during full-blown drug resistance, as it would increase the window during which the vulnerability could be exploited. Such a “collateral sensitivity” approach has been successfully used to target drug-tolerant persister cells [30] as well as cells that have progressed to full-blown drug resistance [81]. Accordingly, the combination of multiple drugs is the number one strategy to prevent resistance to anti-cancer therapy [82,83,84].

Different strategies can be conceived to target DTPs. Targeting epigenetic modifiers, such as the lysine demethylase KDM5, has been shown to decrease the number of persister cells in melanoma, colon and breast cancer models [85]. Likewise, chromatin remodeling controlled by KDM6 and NOTCH signaling has also been shown to promote survival of persister glioblastoma stem-like cells upon receptor-tyrosine kinase inhibition [26]. Targeting the tumor microenvironment can also be a valuable strategy. *BRAF*-mutated melanoma cells initially respond to BRAF inhibition but rapidly become tolerant when in close contact with stromal cells. Mechanistically, melanoma-associated fibroblasts respond to BRAFi with an augmented fibronectin production that enhances FAK signaling in melanoma cells. The coinhibition of BRAF and FAK prevented the reactivation of MAPK and resulted in better control of tumor growth [86]. The altered metabolic and transcriptional state of DTPs can also be exploited for their eradication [6,30]. In addition, the new cell identity adopted by persister cells can be specifically targeted. MAPK inhibition can induce a stem-like transcriptional program driven by RXRG in melanoma cells. RXR antagonism mitigates the accumulation of DTPs and delays the development of resistance [54]. 

An alternative approach could be to redirect DTPs to a permanent and more homogeneous dormant state. Ideally, DTPs should remain dormant for a long period of time so they can be eradicated by the immune system or by targeting specific vulnerabilities. In particular, drug-induced cancer senescence and the consequent SASP activation have been shown to promote immune-mediated clearance of tumor cells in mouse models of lung and pancreatic cancer [87,88]. Furthermore, our group recently published a proof-of-concept where induction of senescence with CDC7 inhibitor in hepatocellular carcinoma models, induces sensitization to mTOR inhibition [89]. Ideally, a therapeutic intervention would simultaneously allow achieving tumor mass shrinkage and prevent the emergency of DTP cells. That this is possible was recently demonstrated: inhibition of CDK7/12, together with inhibition of receptor tyrosine kinases, prevents the appearance of DTPs by blocking the acquisition of transcriptional plasticity needed for survival in response to targeted-therapy [90]. 

The most straightforward therapeutic approach to deal with DTP generation would be to simultaneously target both the oncogenic signaling and the stress response pathways that help cancer cells deal with the oncogenic activity. Because the increased dependence of cancer cells on stress response pathways is intimately linked to the oncogenic activity, perturbation of oncogenic signaling with drugs can improve the efficacy of targeting stress pathways. Experimental data pointing in this direction can be found in the recent literature. For instance, therapy resistance of AML stem cells was reversed by cytokine stimulation, sensitizing these cells, but not normal hematopoietic stem cells, to chemotherapy [91]. It is noteworthy that high DUSP6 level is a predictor of poor clinical outcome for B-ALL patients. In these cells, DUSP6 inhibition and consequently higher levels of ERK1/2 trigger oxidative and replication stress, creating targetable vulnerabilities. Interestingly, patient-derived drug-resistant B-ALL cells showed increased sensitivity to DUSP6 inhibition [92]. Similarly, dormant T-cell Acute Lymphoblastic Leukemia (T-ALL) cells showed high levels of phosphorylated ERK1/2 and p38 and low levels of DUSP1. The DUSP1 protein level is positively regulated by NOTCH3 activity, promoting an aggressive phenotype, while DUSP1 inhibition sensitizes to several chemotherapy drugs [93]. 

The data reviewed here suggest that a better conceptual and mechanistic understanding of the “push and pull” dynamic between oncogenic signaling and stress response pathways may provide a wealth of innovative targeting opportunities to forestall drug tolerance and resistance. 

## 5. Conclusions

In spite of the fact that the interest in the biology of DTPs is relatively recent, a large number of studies have been performed to characterize chemo- and targeted therapy-tolerant persister cells. However, further studies will be required in particular to investigate DTPs in vivo in their crosstalk with the microenvironment and cells of the immune system.

To investigate the vulnerabilities of slow-proliferating drug-tolerant cells, it might be worthwhile to perform unbiased loss of function genetic screens to identify genes essential for their survival. This could lead to the identification of compounds that can be used to kill drug-tolerant cells before they give rise to fully resistant tumors. However, the experimental set-up should take into consideration the low frequency of these cells, which may vary according to tumor type and drug treatment. Most likely, genome-wide screens will be not feasible, and focused libraries should be used for these studies. The fact that different drug-resistant clones can arise from a DTP population may be an additional complicating factor [94,95]. Notwithstanding these technical challenges, a better understanding of the vulnerabilities of DTPs would facilitate strategies to target them upfront, delaying or possibly eliminating the possibility of acquired resistance. Therefore, further development of technologies allowing the single-cell resolution dynamic tracking and genomic profiling of this elusive cell population will be fundamental for the field.
